# Preoperative planning by osteotomy master software helps to improve the accuracy of target limb alignment in high tibial osteotomy

**DOI:** 10.1186/s13018-020-02033-6

**Published:** 2020-11-02

**Authors:** Axiang He, Yanjie Mao, Ying Zhou, Qin Kong, Hui zhang, Yanan Chen, Wanjun Liu, Xianlong Zhang

**Affiliations:** 1 Department of Joint Surgery, Shanghai No. 6 People’s Hospital East Campus, Shanghai Health Medical College, Shanghai, China; 2 Department of Joint Surgery, Shanghai No. 6 People’s Hospital East Campus, Shanghai Jiao Tong University, No. 222, West Huanhu Third Road, Pudong New Area, Shanghai, 201306 China

**Keywords:** Medial compartment osteoarthritis, High tibial osteotomy, Varus deformity, Limb alignment, OsteoMaster

## Abstract

**Background:**

The accuracy of targeted lower limb alignment correction following HTO is closely related to patients’ pain relief and knee joint survival time. How to accurately perform osteotomy and how to obtain the ideal target limb alignment to maximize the curative effect are the difficulty in HTO practice. The purpose of this study is to evaluate the predictive and application value of osteotomy master software (OsteoMaster) in coronal plane preoperative planning of high tibial osteotomy.

**Method:**

Sixty-seven patients with medial compartment osteoarthritis and varus deformity treated by medial open-weight high tibial osteotomy were enrolled and divided into observation group (31 cases) and control group (36 cases). The observation group was planned by OsteoMaster, while the control group was planned by Miniaci. The preoperative predicted values of osteotomy depth, open height, correction angle, WBL ratio, and FTA of the observation group were compared with the actual intraoperative values to study their accuracy. The operative time, blood loss, number of fluoroscopy, and WBL ratio were compared between the observation group and the control group to study its application value.

**Result:**

There was no significant difference between two groups in preoperative prediction and intraoperative reality of osteotomy depth, open height, correction angle, FTA, and WBL ratio (*P* > 0.05). The operation time and number of fluoroscopy in the observation group were significantly less than those in the control group (*P* < 0.05), while the difference in blood loss was not statistically significant (*P* > 0.05). The good rate of WBL ratio was 87.1% in the observation group and 75% in the control group.

**Conclusion:**

OsteoMaster has predictive value in osteotomy depth, open height, correction angle, FTA, and WBL ratio of HTO, which is also helpful to reduce the number of fluoroscopy, shorten the operation time, and improve the accuracy of target limb alignment. The drawback of this approach is 2-dimensional approach in contrast to 3-dimensional preoperative planning that is including the more real study.

## Background

With the aggravation of population aging, the incidence of knee osteoarthritis (OA) increases year by year. It is reported that 4.1 million people over the age of 45 suffer from OA in the UK, while 9.3 million in the USA [1]. When OA enters the terminal stage, total knee replacement (TKA) is an effective treatment to alleviate pain, extend walking distance, and improve patients’ quality of life. However, patients cannot engage in strenuous exercise after TKA, and the prosthesis has a certain number of years of use. For young patients, the surgical effect is limited and the risk of surgical failure increases [2, 3]. Thus, TKA is too aggressive and HTO is an ideal choice for younger patients who have a high demand for activity and are confined to medial compartment osteoarthritis of the knee combined with varus deformity [2, 4].

HTO can release pain, delay the progression of OA, and even create an environment to promote the regeneration of worn cartilage by correcting varus deformity of lower limbs, shifting the weight-bearing line of lower limbs outward, and then dispersing the stress concentration of the medial compartment [5, 6]. HTO is generally suitable for patients younger than 60 years old, with limited medial compartment osteoarthritis, no obvious wear on the tibiofemoral and patellofemoral articular surfaces, tibial varus deformity between 5° and 20°, and high demand for postoperative activity [7, 8]. It has been reported that some athletes can return to the arena after HTO [2].

The accuracy of targeted lower limb alignment correction following HTO is closely related to patients’ pain relief and knee joint survival time [5, 9]. How to accurately perform osteotomy and how to obtain the ideal target limb alignment to maximize the curative effect are the difficulty in HTO practice. The classical limb alignment decision procedure depends on repeated fluoroscopy with a metal rod, which keeps the metal rod passing through the center of the femoral head and ankle joint simultaneously, and observing its position in the knee joint to assess the target limb alignment. However, this method is inefficient [10]. In recent years, the application of a computer navigation system and 3D printing technology has greatly improved the accuracy of osteotomy and shortened the operation time, but the computer navigation machine is expensive and difficult to be widely popularized. The 3D printing technology requires additional CT examination of the hip and ankle joints, and it takes time to make the osteotomy guide plate [5, 11]. This study intends to report a simple and effective method to improve the accuracy of osteotomy and facilitate its popularization and application in grass-roots hospitals with general conditions.

## Materials and methods

### Patients data

A total of 67 patients who underwent medial open-wedge high tibial osteotomy from January 2018 to June 2020 in our hospital were included. Inclusion criteria were as follows: (1) According to the patient’s symptoms, signs, X-ray, and MRI examinations, osteoarthritis of the medial compartment of the knee joint with varus deformity was diagnosed. (2) No or mild osteoarthritis in the tibiofemoral and patellofemoral joints. (3) Varus deformity originates from the tibial side with a range of 5-20°. (4) Range of knee joint motion ≥ 90°, flexion contracture ≤ 10°. (5) The patients who are younger than 60 years. Exclusion criteria: (1) Patients with rheumatoid arthritis, gout arthritis, ankylosing spondylitis, and other autoimmune diseases. (2) Patients with knee joint tumor, infection, tuberculosis, dwarf discase, severe osteoporosis, and other diseases affecting osteotomy healing.

### OsteoMaster

**Fig. 1:**
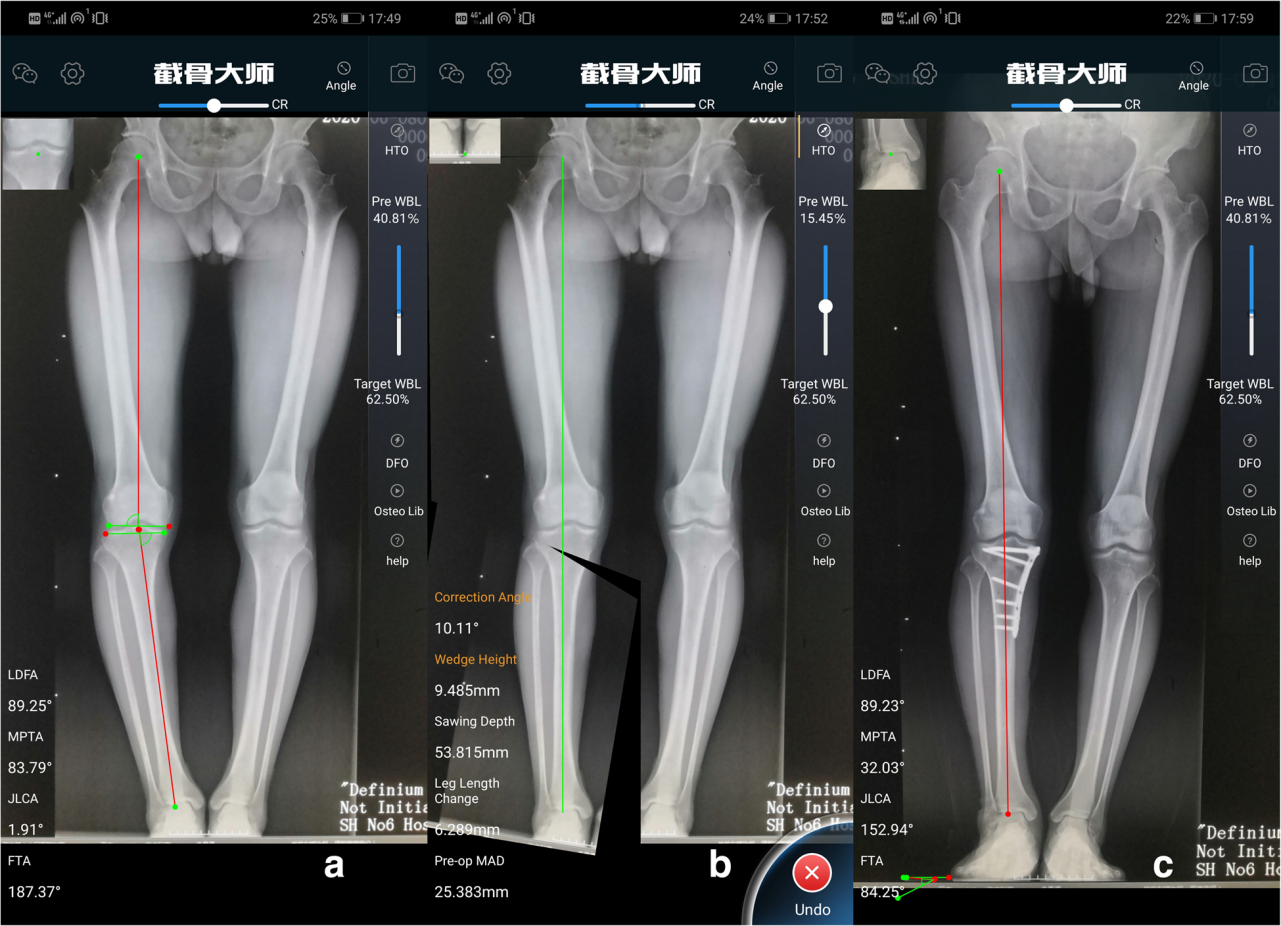
A 54-year-old male who suffers from right medial compartment osteoarthritis with varus deformity for 4 years. Preoperative measurements by OsteoMaster: FTA 187.4°, MPTA 83.8°, LDFA 89.3°, JLCA 1.9° (**a**). Preoperative prediction: osteotomy depth 53.8 mm, open height 9.5 mm, correction angle 10.1°, WBL 62.5% (**b**). Actual value during operation: osteotomy depth 51.6 mm, open height 10 mm, WBL 60.9%, FTA 177.3°, correction angle 10.1° (**c**)

OsteoMaster is a mobile application (APP) that can be downloaded after purchased from the APP store. Its main function is to carry out preoperative planning according to the scale, predict osteotomy parameters such as depth of osteotomy, open/closed height, correction angle, on the basis of weight-bearing full-length X-ray radiography of lower limbs. OsteoMaster is able to carry out simulated osteotomy and is suitable for preoperative planning of HTO and distal femur osteotomy (DFO) (Fig. 1).

### Grouping and observation indicators

According to the random number, the patients were divided into observation group (odd array) 31 cases and control group (even array) 36 cases. There were no significant differences in age, gender, preoperative FTA, MPTA, and LDFA between the two groups (Table 1). The weight-bearing full-length X-rays of lower limbs which were taken with 15 degrees of internal rotation; CT and MRI scan of knee joints were completed in both groups at admission. In the observation group, the varus angle, MPTA, and LDFA were measured on the full-length radiography of lower limbs by OsteoMaster, which was then used for preoperative planning to predict the osteotomy depth, open height, correction degree, and post-operative FTA when weight-bearing line (WBL) ratio equals to 62.5%. Among them, the postoperative FTA and the WBL ratio were measured in full-length radiography of lower limbs at the weight-bearing position which was performed after surgery. The predicted value was compared with the actual occurrence value to analyze the prediction accuracy.

**Table 1:** Comparison of baseline between observation group and control group

	Cases	Gender (M/F)	Age	FTA (°)	LDFA (°)	MPTA (°)
**Observation group**	31	18/13	56.3 ± 8.8	187.4 ± 3.4	87.9 ± 3.0	82.1 ± 2.7
**Control group**	36	24/12	55.0 ± 9.2	188.8 ± 3.0	86.6 ± 2.7	82.8 ± 2.3
***t*/*χ***^**2**^		0.527	0.588	1.791	1.867	1.146
***P***		0.468	0.558	0.078	0.067	0.256

In the control group, the angle of correction is determined by Miniaci [12]. The distance from the center of the ankle joint to hinge was taken as the radius to rotate until it intersected with target limb alignment. The angle of rotation was the angle to be corrected. During the operation, the depth of osteotomy was determined according to the results of Kirschner-wire insertion and the height of osteotomy was calculated using triangular function. The differences between OsteoMaster and Miniaci prediction in operation time, blood loss, number of fluoroscopy, and target limb alignment were compared to evaluate the application value of OsteoMaster. According to the research of Fujisawa, it was defined as good when WBL is located at 55-70% and poor when WBL is more than 70% or less than 55%.

### Surgical technique

All patients underwent open-wedge high tibial osteotomy to correct varus deformity. After routine disinfection, toweling, and exposure, two Kirschner wires were implanted 3-4 cm below the medial tibial plateau, pointing in the direction of the fibula head, and fluoroscopy was performed to determine the location. Patients in the observation group were osteotomized according to the prediction of OsteoMaster, while patients of the control group were guided to osteotomy according to Miniaci planning. The metal rod was used for target limb alignment determination when fluoroscopy during operation by repeatedly adjusting until it falls at the Fujisawa point and passes through the center of the femoral head and ankle joint at the same time. Then, TOMOFIX was used for strong internal fixation after allograft bone was implanted in the tibial gap. The incision was then sterile and a negative pressure drainage tube was placed. All patients moved down with the assistance of walking aids and weight-bearing on the second day after surgery. Lower limb weight-bearing full-length radiography was taken to estimate the accuracy of limb alignment.

### Statistical analysis

SPSS 18.0 was used for statistical analysis. Age, FTA, MPTA, LDFA, open height, and osteotomy depth were all measured data and belonged to continuous variables. Statistical results were expressed as (‾X ± S). Two independent samples *t* test was used for statistical analysis. Gender and ratio were counted data, and statistical analysis was performed by chi-square test or Fisher’s exact probability method. *P* < 0.05 indicated that the difference was statistically significant.

## Result

### Comparison of preoperative predicted and intraoperative actual value in the observation group

The preoperative predicted value and intraoperative actual value were no statistically significant differences in osteotomy depth, open height, correction angle, target FTA, and WBL ratio (*P* > 0.05) (Table 2).

**Table 2:** Comparison of preoperative predicted and intraoperative actual values in the observation group

	Depth (mm)	Open weight (mm)	WBL ratio	FTA (°)	Correction angle (°)
**Preoperative prediction**	53.4 ± 6.5	9.8 ± 2.9	0.625 ± 0.00	175.8 ± 2.5	11.6 ± 2.2
**Actual values**	50.6 ± 7.2	11.0 ± 2.5	0.647 ± 0.09	174.7 ± 2.5	12.7 ± 2.4
***t*/χ**^**2**^	1.607	1.745	1.361	1.839	1.881
***P***	0.113	0.086	0.179	0.071	0.065

### Comparison of operation indexes between the observation group and the control group

The operation time and number of fluoroscopy in the observation group were significantly less than those in the control group (*P* < 0.05). There was no significant difference in blood loss between the two groups (*P* > 0.05). Patients with WBL ratio between 55 and 70% accounted for 87.1% in the observation group and 75% in the control group. There were no serious complications during the operation in both groups (Table 3).

**Table 3:** Comparison of operation parameters between the observation group and control group

	Operating time (min)	Blood loss (ml)	Number of fluoroscopy	WBL accuracy (%)
**Observation group**	51.2 ± 10.0	57.7 ± 13.7	5.3 ± 2.2	87.1%
**Control group**	62.0 ± 11.4	64.3 ± 15.2	9.5 ± 3.1	75.0%
***t*/*χ***^**2**^	4.090	1.854	6.298	
***P***	0.001	0.068	< 0.0001	

## Discussion

As a classic knee preserving surgery, HTO has developed for nearly 60 years and is one of the effective surgical therapies for the medial compartment osteoarthritis of the knee with varus deformity [13, 14]. After knee varus, the stress of lower limbs is concentrated in the medial compartment of the knee in the weight-bearing position, resulting in the increase of pressure on the cartilage surface. When the bearing capacity of the cartilage is exceeded, articular cartilage damage would occur, leading to osteoarthritis, knee pain, and limited activity, and affecting the patient’s quality of life. At the same time, because the pressure of the medial compartment continues to rise, the medial joint space gradually narrows, which easily leads to the relaxation of the lateral collateral ligament and the tightening of the medial collateral ligament, thus further aggravating the varus deformity, aggravating osteoarthritis and forming a vicious circle [14–16]. When varus deformity mainly comes from the side of the tibia, HTO, by correcting the mechanical axis of the lower limbs, shifts the weight-bearing line outward and alleviates the stress concentration state in the medial compartment, which can effectively block the vicious cycle, improve the function of the knee joint, and delay the degeneration of cartilage to the greatest extent [17, 18].

The clinical efficacy and indication selection of HTO are closely related to the accuracy of the target limb alignment [17, 19]. Although the choice of indications is still controversial, the vast majority of scholars believe that HTO has a good effect in younger patients who are limited to medial compartment osteoarthritis, do not involve bone-to-bone wear, combine with mild and moderate varus deformity originating from the tibial side, and have a greater demand for mobility [20, 21]. Most scholars believe that [14, 22–24] the weight-bearing line should be corrected to slightly valgus, so as to effectively reduce the medial compartment stress and avoid the recurrence of varus deformity, but excessive valgus will increase the lateral compartment stress, which has the risk of causing lateral compartment osteoarthritis. According to different literature reports [1, 7], good long-term efficacy can be achieved when correction is made to valgus at 3-13°. Fujisawa et al. [25] showed that 30-40% of the lateral WBL ratio could achieve a better effect, and the best effect was achieved when WBL ratio reach to 62.5%.

Target limb alignment can affect long-term survival rate of the knee joint. However, how to obtain accurate target limb alignment is a common problem faced by joint surgeons. Prediction of FTA correction would be done by Miniaci way in traditional preoperative planning and intraoperative fluoroscopy with metal rods is used to find the midpoint of the hip and ankle joints to determine the location of WBL [12]. The direction of fluoroscopy and the position of the patient will have an impact on the accuracy of the results. To adjust the limb alignment, multiple fluoroscopy is needed to find the center of the hip and ankle joint, which prolongs the operation time and increases the damage of the radiation to the patients and doctors [26, 27]. The use of computer navigation technology can not only display the change of FTA angle dynamically in real-time but also observe the intersection position of the mechanical axis of lower limbs at the knee joint, which greatly improves the osteotomy accuracy and shortens the operation time. However, due to the different tension of muscles under awake and anesthesia at the knee joint, the mechanical axis of lower limbs may still have a slight deviation after surgery [5, 28]. Accurate osteotomy can also be achieved by the use of a 3D-printed osteotomy guide plate, which can be fabricated according to the data of hip, knee, and ankle CT scan. However, this process increases the patient’s economic burden, radiation damage, and prolongs the time of preoperative preparation [11]. If there is a more concise means to improve the accuracy of the target limb alignment without additional equipment and economic burden, it will contribute to the popularity of the operation.

In our study, it was found that preoperative planning by OsteoMaster was simple and effective to improve the accuracy of the target limb alignment. The preoperative planning can be carried out according to the weight-bearing full-length radiography of lower limbs and its scale, without additional multi-position CT scan or using expensive and difficult-to-obtain equipment. Our study found that although the preoperative predictive values of osteotomy depth, open height, angle of correction, target FTA, WBL ratio, and intraoperative actual values of 31 patients had a slight discrepancy, there was no statistical significance, which indicated the accuracy of preoperative planning of OsteoMaster. Further study found that preoperative planning with OsteoMaster can help shorten the operation time, reduce the number of intraoperative fluoroscopy, and increase the proportion of WBL to 87.1%, which has a certain application value. The results of our study showed that the reduction of operation time did not cause a decrease in the amount of blood loss, possibly because the amount of blood loss was low after the application of lower limb tourniquet. All the patients in this study had no complications such as hinge fracture and intra-articular injury, confirming the use of OsteoMaster did not increase the risk.

This study intends to evaluate the application value of OsteoMaster in HTO preoperative planning, and preliminarily prove its effectiveness and safety, but this study has some shortcomings. HTO should not only reconstruct the physiological mechanical axis of lower limbs in the coronal plane but also reconstruct or maintains the original physiological parameters in sagittal and horizontal positions. The OsteoMaster can only be used to correct the coronal limb alignment, but cannot predict the tibial rotation and sagittal tibial retroversion after surgery. Effective preoperative planning for tibial and femoral complex varus deformity is not possible. The OsteoMaster makes measurement based on X-ray, which is affected by the patient’s position and exposure distance, so that it may be easy to cause errors.

## Conclusion

OsteoMaster based on standard radiography of lower limb weight-bearing full-length X-rays radiography has a certain predictive value for coronal intraoperative and postoperative related parameters of HTO, which can improve the accuracy of target limb alignment, shorten the operation time, and reduce the number of fluoroscopy, without relying on additional imaging examination and expensive equipment. However, the drawback of this approach is 2-dimensional approach in contrast to 3-dimensional preoperative planning that is including the more real study. The parameters of horizontal and sagittal plane, such as tibial rotation and slope, cannot be preoperatively planned, and it still needs further improvement.

## Data Availability

The datasets during and/or analyzed during the current study available from the corresponding author on reasonable request.

## Abbreviations

OA: Osteoarthritis
HTO: High tibial osteotomy
FTA: Femorotibial angle
LDFA: Lateral distal femoral angle
MPTA: Medial proximal tibia angle
DFO: Distal femur osteotomy
OsteoMaster: Osteotomy master software
